# A Population-Based Cohort Study on Efficacy and Safety of Bariatric Surgery in Young Adults Versus Adults

**DOI:** 10.1007/s11695-023-06673-5

**Published:** 2023-06-26

**Authors:** Kelly G. H. van de Pas, Aliyar Esfandiyari Noushi, Loes Janssen, Anita C. E. Vreugdenhil, Wouter K. G. Leclercq, François M. H. van Dielen, Dutch Audit for Treatment of Obesity Research Group, G. J. D. van Acker, J. A. Apers, F. Berends, L. M. de Brauw, F. F. E. Bruinsma, S. M. M. de Castro, S. L. Damen, F. Jonker, I. F. Faneyte, J. W. M. Greve, G. van ’t Hof, R. A. Klaassen, E. A. G. L. Lagae, B. S. Langenhoff, R. S. L. Liem, A. A. P. M. Luijten, S. W. Nienhuijs, R. M. Smeenk, S. J. M. Smeets, W. Vening, M. J. Wiezer, E. de Witte

**Affiliations:** 1 Department of Surgery, Máxima Medical Center, 5504DB Veldhoven, The Netherlands; 2 Department of Pediatrics, Maastricht University Medical Center, 6220HX Maastricht, The Netherlands; 3 NUTRIM School of Nutrition and Translational Research in Metabolism, Maastricht University, 6229 ER Maastricht, The Netherlands

**Keywords:** Bariatric surgery, Young adults, Adults, Gastric bypass, Gastric sleeve

## Abstract

**Purpose:**

Bariatric surgery is the most effective treatment for severe obesity in adults and has shown promising results in young adults. Lack of insight regarding efficacy and safety outcomes might result in delayed bariatric surgery utilization in young adults. Therefore, this study aimed to assess the efficacy and safety of bariatric surgery in young adults compared to adults.

**Methods:**

This is a nationwide population-based cohort study utilizing data from the Dutch Audit Treatment of Obesity (DATO). Young adults (aged 18–25 years) and adults (aged 35–55 years) who underwent primary Roux-en-Y gastric bypass (RYGB) or sleeve gastrectomy (SG) were included. Primary outcome was percentage total weight loss (%TWL) until five years postoperatively.

**Results:**

A total of 2,822 (10.3%) young adults and 24,497 (89.7%) adults were included. The follow-up rates of the young adults were lower up to five years postoperatively (46.2% versus 56.7% three years postoperatively; p < 0.001). Young adults who underwent RYGB showed superior %TWL compared to adults until four years postoperatively (33.0 ± 9.4 versus 31.2 ± 8.7 three years after surgery; *p* < 0.001). Young adults who underwent SG showed superior %TWL until five years postoperatively (29.9 ± 10.9 versus 26.2 ± 9.7 three years after surgery; *p* < 0.001). Postoperative complications ≤ 30 days were more prevalent among adults, 5.3% versus 3.5% (*p* < 0.001). No differences were found in the long term complications. Young adults revealed more improvement of hypertension (93.6% versus 78.9%), dyslipidemia (84.7% versus 69.2%) and musculoskeletal pain (84.6% versus 72.3%).

**Conclusion:**

Bariatric surgery appears to be at least as safe and effective in young adults as in adults. Based on these findings the reluctance towards bariatric surgery in the younger age group seems unfounded.

**Graphical Abstract:**

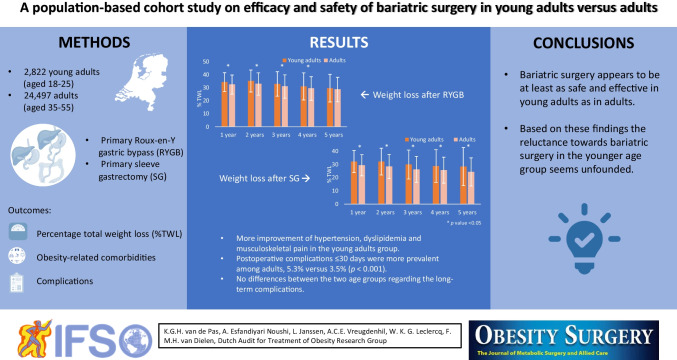

**Supplementary Information:**

The online version contains supplementary material available at 10.1007/s11695-023-06673-5.

## Introduction

Bariatric surgery has proven to be the most effective treatment for severe obesity in adults and has shown promising results in young adults in achieving weight loss and remission of obesity related comorbidities [1, 2]. A potential benefit of bariatric surgery at a younger age is a shorter exposure to obesity related comorbidities, such as type 2 diabetes mellitus (T2DM), which can lead to less medical complications and treatment resistance [3, 4]. It might also limit the obesity related psychosocial ‘challenges’ such as bullying and stigmatizing at this vulnerable age [5]. However, bariatric surgery utilization in young adults lags behind. This might among others be attributed to a lack of understanding regarding efficacy and safety outcomes in this younger age group [6]. The amount of young adults that underwent bariatric surgery in the United States even slightly decreased in the last couple of years. In 2006 22.4% of all patients who underwent bariatric surgery were young adults compared to 20.3% in 2015 [7].

Prior research has mainly focused on the efficacy and safety of bariatric surgery in the whole group of adults. Only a small amount of studies addressed the effects of bariatric surgery in the younger age group. Many of these studies targeted adolescents (generally ≤ 19 years old) and revealed at least comparable results when it comes to weight loss, and equal or more surgery related complications compared to adults [8, 12]. In line with this, a Swedish national registry study among 3,531 young adults (aged 18–25 years) and 17,137 adults (aged ≥ 26 years) observed superior weight loss in young adults compared to adults up to five years postoperatively. Besides this, serious adverse events, defined as a Clavien-Dindo ≥ 3b, were more prevalent among young adults between 6 weeks and up to 5 years after a Roux-en-Y gastric bypass (RYGB) [13]. Young adults and adults who underwent a sleeve gastrectomy (SG) were excluded from the study, despite it being the most frequently performed bariatric procedure worldwide along with the RYGB [14].

In order to obtain a better understanding on the efficacy and safety of bariatric surgery in the younger age group, this study aimed to compare weight-related outcomes and complications between young adults (aged 18–25 years) and adults (aged 35–55 years) who underwent a RYGB or SG.

## Methods

### Study Design

This nationwide population-based cohort study was conducted with pseudo-anonymized data derived from the Dutch Audit Treatment of Obesity (DATO). The DATO is a national mandatory registry for all bariatric procedures performed in the Netherlands starting in 2015 and including all 20 bariatric surgical centres [15]. The Institutional Review Board of the Máxima MC approved this study. Patient consent was not required for this retrospective cohort study according to Dutch law (Medical Research Involving Human Subjects Act).

### Setting and Participants

Eligibility for bariatric surgery in the Netherlands is assessed according to the Dutch guideline surgical treatment of obesity [16]. The bariatric surgery candidates are evaluated by a multidisciplinary team and need to be ≥ 18 years and have a pre-operative body mass index (BMI) ≥ 40 kg/m^2^ or a BMI ≥ 35 kg/m^2^ accompanied by an obesity-related comorbidity*.* Young adults (aged 18–25 years) and adults (aged 35–55 years) who received a primary RYGB or SG between 2015 and 2020 were screened for inclusion in this study. A time range of 10 years around the mean age of adults undergoing bariatric surgery (45 years) was chosen as the control group for the young adults [17]. Participants were excluded when they underwent a two-stage procedure, had a missing body weight 12 months after surgery or had a BMI < 35 kg/m^2^.

### Outcome Parameters – Weight Loss

Outcome parameters were collected at baseline and one to five years (± three months) after surgery during outpatient clinic visits. Percentage TWL one to five years after surgery was the primary outcome and was calculated using the following formula: (preoperative weight-postoperative weight)/preoperative weight * 100%. Secondary outcomes were successful weight loss and weight regain. Percentage TWL ≥ 20% was considered as successful weight loss [18, 19]. Weight regain was defined as ≥ 20% regain of a patients’ lost weight at their last follow-up visit after initial successful weight loss one year after surgery [18, 19].

### Outcome Parameters – Complications

Complications were divided into perioperative complications, postoperative complications ≤ 30 days and postoperative complications > 30 days. The Clavien-Dindo (CD) classification of surgical complications was used to classify the postoperative complications [20]. In case of multiple complications in one patient, only the highest CD complication was used in the CD and readmission analyses. In the other analyses all complications were used.

### Outcome Parameters – Comorbidities

Secondary outcomes included the obesity related comorbidities, e.g. T2DM, hypertension, dyslipidemia, gastroesophageal reflux disease (GERD), obstructive sleep apnea (OSA), and musculoskeletal pain. The postoperative comorbidity status was classified as cured or improved, and equal or worsened according to the DATO, and compared to the preoperative comorbidity status [21]. The comorbidity status of one and two years after surgery was combined, the last status was chosen. This outcome was only assessed up to two years postoperatively, as the comorbidity status was frequently missing at three, four and five years after surgery which led to small numbers in the young adults group.

### Statistical Analysis

Statistical analyses were conducted using IBM SPSS statistic software, version 25.0, Armonk, NY. Numerical data (baseline characteristics, weight loss) were analyzed using independent-samples t-test and presented as mean ± standard deviation (SD). Categorical data (baseline characteristics, complications, revision procedures and obesity related comorbidities) were analyzed using chi-square test and presented as number (percentage). Linear mixed model (LMM) analysis was used to assess %TWL one to five years after surgery between adults and young adults. In this model the factor-analytic covariance matrix and restricted maximum likelihood estimation were utilized. In the model, an interaction variable for age category and bariatric procedure was added, since %TWL might depend on the type of bariatric procedure. In case of a significant interaction, the effect of age category was presented separately for RYGB and SG. Furthermore, corrections were made based on the variables having a significant association with %TWL in univariate analyses. At last, a sensitivity analysis was performed in which %TWL was only assessed up to three years after surgery due to the large lost to follow-up four and five years after surgery. In a second sensitivity analysis, the %TWL until three years after surgery was compared between participants with four- and five-year follow-up data and participants with only one-to-three-year follow-up data using an independent-samples t-test. All data were analyzed according to the intention to treat principle and *p* values ≤ 0.05 were considered statistically significant.

## Results

**Fig. 1:**
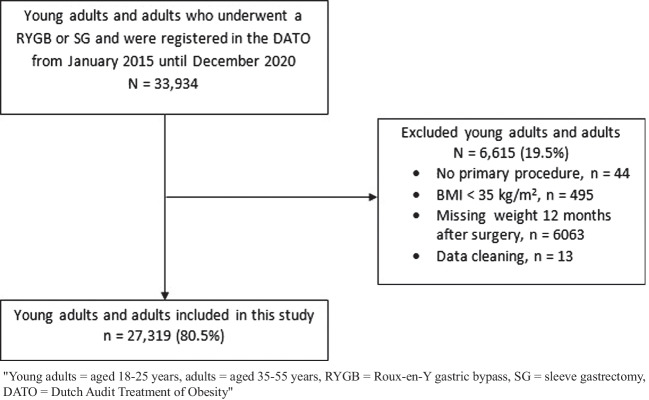
Flow diagram of the inclusion and exclusion of participants

A total of 33,934 young adults and adults underwent a primary RYGB or SG between 2015 and 2020 in the Netherlands. Of them 27,319 (80.5%) were included, 925 young adults (24.7%) and 5690 adults (18.8%) were excluded due to various reasons such as a BMI < 35 kg/m^2^ or a missing weight 12 months after surgery (Fig. 1). In this study 2,822 (10.3%) young adults and 24,497 (89.7%) adults were included. No clinically relevant differences were found between the in- and excluded young adults and adults regarding their age, gender, BMI and obesity related comorbidities (data not shown). Compared to the adults, the follow-up rates of the young adults were lower (all *p* < 0.001). Two through five-year follow-up rates in the young adults group were 62.9%, 46.2%, 36.8% and 28.3%, respectively. Whereas in the adult group, two through five-year follow-up rates were 71.4%, 56.7%, 45.8% and 38.4%.

### Baseline Characteristics and Operative Features

In the young adults group, there were more females compared to the adults group, 87.0% versus 79.1% (*p* < 0.001). Furthermore, the preoperative BMI was higher in the young adults, whereas all the preoperative comorbidities were more prevalent among the adults (Table 1). In 2015 only 8.6% of the bariatric procedures were performed in young adults, this increased over time and became 12.3% in 2020. Young adults more often underwent a SG compared to adults (45.8% versus 22.9%; *p* < 0.001). The number of young adults undergoing bariatric surgery increased by age year. Only 2.3% (*n* = 66) of the young adults were 18 years old, whereas 20.1% (*n* = 567) were 25 years old. On the contrary, the mean BMI of the young adults who underwent bariatric surgery generally decreased by age year. The mean BMI of the 18-year-olds was 46.0 ± 5.8 kg/m^2^, whereas the mean BMI of the 25-year-olds was 44.5 ± 5.2 kg/m^2^. This trend regarding number of bariatric procedures and BMI was not observed in the adults group.

**Table 1:** Baseline characteristics and operative features of the included young adults and adults

	Young adults, *n* = 2,822 (10.3%)	Adults, *n* = 24,497 (89.7%)	*P* value
*Age (years,* ± *SD)*	22.5 ± 2.0	45.9 ± 5.8	< 0.001*
*Sex, n. (%)* *Female* *Male*	2,455 (87.0) 367 (13.0)	19,381 (79.1) 5,116 (20.9)	< 0.001*
*Preoperative BMI (kg/m*^*2*^*,* ± *SD)*	44.4 ± 4.9	42.8 ± 5.2	< 0.001*
*Preoperative comorbidities, n. (%)* *T2DM* *Hypertension* *Dyslipidemia* *GERD* *OSA* *Musculoskeletal pain*	97 (3.4) 137 (4.9) 145 (5.1) 248 (8.8) 119 (4.2) 881 (31.2)	4,510 (18.4) 8,709 (35.6) 4,761 (19.4) 4,012 (16.4) 4,508 (18.4) 11,140 (45.5)	< 0.001* < 0.001* < 0.001* < 0.001* < 0.001* < 0.001*
*Year of surgery, n. (%)*^*1*^ *2015* *2016* *2017* *2018* *2019* *2020*	394 (8.6) 483 (9.3) 508 (10.7) 510 (10.7) 525 (11.0) 402 (12.3)	4,203 (91.4) 4,704 (90.7) 4,218 (89.3) 4,241 (89.3) 4,257 (89.0) 2,874 (87.7)	< 0.001*
*Bariatric procedure, n. (%)* *RYGB* *SG*	1,529 (54.2) 1,293 (45.8)	18,890 (77.1) 5,607 (22.9)	< 0.001*
*Length of hospital stay in days [Q1, Q3]*	1 [1, 2]	1 [1, 2]	0.632

Data presented as number (%), mean (± SD) or median [Q1, Q3]

*p value is below the threshold of ≤ 0.05

Young adults = aged 18–25 years, adults = aged 35–55 years, *n* = number, BMI = body mass index, T2DM = type 2 diabetes mellitus, GERD = gastroesophageal reflux disease, OSA = obstructive sleep apnea, RYGB = Roux-en-Y gastric bypass, SG = sleeve gastrectomy

^1^(number of operated young adults or adults/total number of bariatric procedures during that year) * 100

### Weight Loss

Both when young adults underwent a RYGB or SG, %TWL was superior compared to the %TWL of adults one to five years after surgery (Table 2). In a sensitivity analysis, in which %TWL until three years postoperatively was compared between participants with four- and five-year follow-up data and participants with only one-to-three-year follow-up data, similar results were found. Furthermore, successful weight loss (≥ 20% TWL) was more prevalent among young adults one to two years after surgery in the ones who received a RYGB and one to three years after surgery in the ones who received a SG (Table 2). Weight regain occurred in 148 young adults (15.7%) and 2,351 adults (17.6%; *p* = 0.136) who underwent a RYGB, and in 175 young adults (21.2%) and 1,038 adults (26.3%; *p* = 0.002) who underwent a SG.

**Table 2:** Percentage TWL and successful weight loss between young adults and adults who underwent a primary RYGB or SG

	Young adults		Adults		*P value*
	*RYGB*		*RYGB*		
*TWL*	n	% ± SD	n	% ± SD	
*1 year* *2 years* *3 years* *4 years* *5 years*	1,529 813 452 280 160	34.4 ± 7.2 35.2 ± 8.4 33.0 ± 9.4 31.0 ± 10.4 29.7 ± 10.6	18,890 11,927 7,622 4,717 2,788	32.6 ± 7.2 33.1 ± 8.3 31.2 ± 8.7 29.6 ± 8.9 28.8 ± 9.2	< 0.001* < 0.001* < 0.001* 0.024* 0.282
*Successful weight loss*^*1*^	n	%	n	%	
*1 year* *2 years* *3 years* *4 years* *5 years*	1,487 776 404 238 134	97.3 95.4 89.4 85.0 83.8	18,143 11,179 6,849 4,048 2,310	96.0 93.7 89.9 85.8 82.9	0.018* 0.048* 0.744 0.704 0.770
	***SG***		***SG***		
*TWL*	n	% ± SD	n	% ± SD	
*1 year* *2 years* *3 years* *4 years* *5 years*	1,293 709 423 230 88	32.1 ± 8.4 32.1 ± 10.1 29.9 ± 10.9 28.7 ± 12.3 28.3 ± 14.4	5,607 3,516 2,221 1,294 636	29.3 ± 8.0 28.4 ± 9.2 26.2 ± 9.8 25.6 ± 9.8 24.3 ± 10.6	< 0.001* < 0.001* < 0.001* < 0.001* 0.012*
*Successful weight loss*^*1*^	n	%	n	%	
*1 years* *2 years* *3 years* *4 years* *5 years*	1,188 626 343 173 63	91.9 88.3 81.1 75.2 71.6	4,948 2,886 1,640 920 407	88.2 82.1 73.8 71.1 64.0	< 0.001* < 0.001* 0.002* 0.201 0.162

Data presented as mean (± SD) and *n* (%)

*p value is below the threshold of ≤ 0.05

Young adults = aged 18–25 years, adults = aged 35–55 years, RYGB = Roux-en-Y gastric bypass, SG = sleeve gastrectomy, TWL = total weight loss, *n* = number

^1^Defined as ≥ 20% TWL

**Fig. 2:**
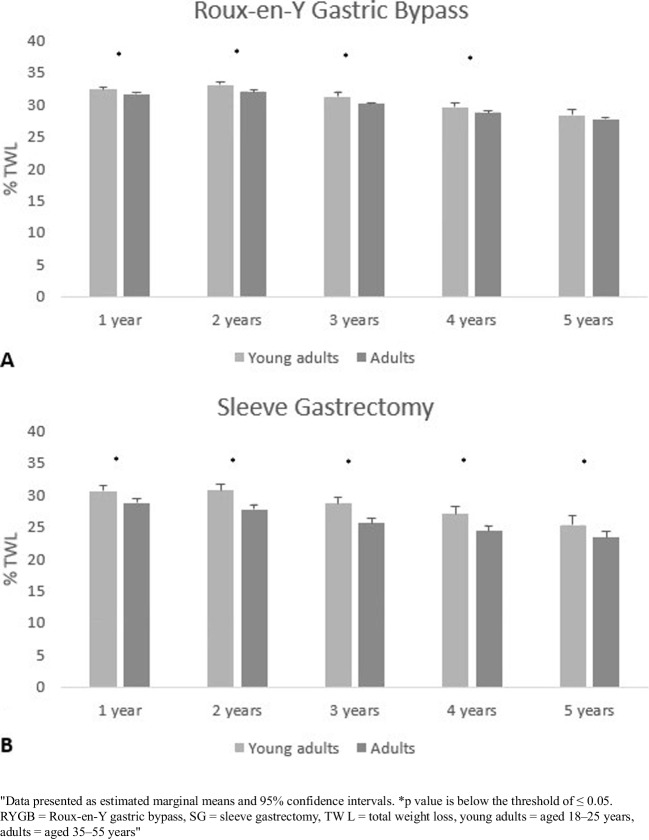
Percentage TWL between young adults and adults until five years after surgery, stratified for bariatric procedure (RYGB = panel **A**, SG = panel **B**) and determined by a linear mixed model (including age category, time and their interaction)

A linear mixed model (LMM) analyzed the association between %TWL and age category (young adults versus adults). These analyses were stratified for bariatric procedure, since age category and bariatric procedure revealed a significant interaction effect on %TWL. Besides this, corrections were made for sex, hypertension, T2DM, OSA, dyslipidemia, BMI and postoperative complications > 30 days as these variables were significantly associated with %TWL in univariate analyses (Appendix Table 1). After adjusting for these variables, LMM revealed a higher %TWL one to four years after surgery for young adults who underwent a RYGB and one to five years after surgery for young adults who underwent a SG (Fig. 2). In a sensitivity analysis, in which %TWL was only assessed until three years after surgery due to the large lost to follow-up four and five years after surgery, similar results were found.

### Complications

As shown in Table 3, more peroperative gastrointestinal perforations were observed in the adults compared to the young adults; 85 (0.3%) versus 1 (0.0%; *p* = 0.005). Furthermore, the total number of postoperative complications and severe postoperative complications (CD ≥ III) ≤ 30 days were higher in the adults group. When looking into the postoperative complications ≤ 30 days, major bleedings and anastomotic leakages were more prevalent among adults compared to young adults; 393 (1.6%) versus 20 (0.7%; *p* < 0.001), and 133 (0.5%) versus 4 (0.1%; *p* = 0.004). No differences between the incidence of postoperative complications > 30 days were found between the two age groups.

**Table 3:** Comparison of per- and postoperative complications between young adults and adults who underwent a primary RYGB or SG

	Young adults, *n* = 2,822	Adults, *n* = 24,497	*P* value
*Peroperative complications, n.(%)* *Perforation* *Bleeding* *Spleen injury* *Liver injury*	19 (0.7) 1 (0.0) 7 (0.2) 3 (0.1) 2 (0.1)	235 (1.0) 85 (0.3) 63 (0.3) 34 (0.1) 32 (0.1)	0.138 0.005* 0.935 1.000 0.575
*Number of readmissions* ≤ *30 days*^*1*^*, n. (%)*	58 (2.1)	595 (2.4)	0.219
*Postoperative complications* ≤ *30 days*^*1*^*, n. (%)* *CD grade I* *CD grade II* *CD grade III* *CD grade IV*	14 (0.5) 23 (0.8) 30 (1.1) 3 (0.1)	192 (0.8) 201 (0.8) 470 (1.9) 116 (0.5)	0.094 0.976 0.001* 0.005*
*Total number of postoperative complications* ≤ *30 days, n. (%)*	98 (3.5)	1,303 (5.3)	< 0.001*
*Type of surgical complications* ≤ *30 days, n. (%)* *Major bleeding* *Anastomotic leakage* *Intra-abdominal abscess* *Intestinal obstruction* *Anastomotic stricture* *Stomach ulcer* *Bowel injury*	20 (0.7) 4 (0.1) 2 (0.1) 8 (0.3) 4 (0.1) 0 (0) 0 (0)	393 (1.6) 133 (0.5) 55 (0.2) 57 (0.2) 15 (0.1) 6 (0.0) 19 (0.1)	< 0.001* 0.004* 0.090 0.600 0.124 NA NA
*Number of readmission* > *30 days*^*1*^*, n. (%)*	120 (4.3)	1,032 (4.2)	0.921
*Postoperative complications* > *30 days*^*1*^*, n. (%)* *CD grade I* *CD grade II* *CD grade III* *CD grade IV*	4 (0.1) 10 (0.4) 119 (4.2) 1 (0)	51 (0.2) 96 (0.4) 1000 (4.1) 8 (0)	0.456 0.761 0.732 0.939
*Total number of postoperative complications* > *30 days, n. (%)*	177 (6.3)	1,512 (6.2)	0.835

Data presented as number (%)

*p value is below the threshold of ≤ 0.05

Young adults = aged 18–25 years, adults = aged 35–55 years, RYGB = Roux-en-Y Gastric Bypass, SG = sleeve gastrectomy, *n* = number, CD = Clavien–Dindo classification, I is any deviation from the normal postoperative course without intervention, except some drugs such as anti-emetics, antipyretics, analgesics, diuretics and electrolytes; II is a complication requiring pharmacological treatment other than such allowed for grade I; III is a complication requiring intervention under anesthesia; IV is a complication resulting in organ failure, NA = not applicable

^1^ = only the highest CD complication has been registered for each patient

### Revision Surgery

A total of 418 young adults and adults underwent revision surgery until December 2021, of which 54 were young adults (1.9%) and 364 were adults at the time of the primary procedure (1.5%; *p* = 0.080). The mean duration between primary and revision procedure was 27.7 months for young adults and 31.0 months for adults. Of the revision procedures 335 were primarily a SG (4.9% of the total number of SG) and 83 a RYGB (0.4% of the total number of RYGB; *p* < 0.001). In the young adult group, the most commonly performed revision procedure was a conversion to a gastric bypass (*n* = 39, 72.2%) and the main reason for revision was technical failure (*n* = 29, 53.7%) followed by weight regain (*n* = 12, 22.2%). In adults, the most frequently executed procedure was a conversion to a gastric bypass (*n* = 230, 63.2%) and the main reasons for revision procedures were also technical failure (*n* = 201, 55.2%) and weight regain (*n* = 64, 17.6%). Furthermore, in the adult group nine adults underwent a second revision procedure and one adult received a third revision procedure, whereas this wat not seen in the young adults.

### Obesity Related Comorbidities

With regard to the regression of obesity related comorbidities one and two year after surgery, the young adults revealed more curation or improvement of hypertension, dyslipidemia and musculoskeletal pain (Table 4). No differences between the two age groups were found in the other obesity related comorbidities.

**Table 4:** The regression of obesity related comorbidities between young adults and adults who received a primary RYGB or SG

	Young adults 1–2 years after surgery			Adults 1–2 years after surgery			
	n. (%)^1^	Cured or improved n. (%)	Equal or worsened n. (%)	n.^1^	Cured or improved n. (%)	Equal or worsened n. (%)	*P *value
*T2DM*	64 (66.0)	57 (89.1)	7 (10.9)	3,714 (82.4)	3,377 (90.9)	339 (9.1)	0.607
*Hypertension*	109 (79.6)	102 (93.6)	7 (6.4)	7,363 (84.5)	5,812 (78.9)	1,551 (21.1)	< 0.001*
*Dyslipidemia*	111 (76.6)	94 (84.7)	17 (15.3)	3,836 (80.6)	2,656 (69.2)	1,180 (30.8)	< 0.001*
*GERD*	143 (57.7)	114 (79.7)	29 (20.3)	1,935 (48.2)	1,588 (82.1)	347 (17.9)	0.482
*OSAS*	69 (58.0)	57 (82.6)	12 (17.4)	3,562 (79.0)	2,983 (83.7)	579 (16.3)	0.800
*Musculoskeletal pain*	571 (64.8)	483 (84.6)	88 (15.4)	7,614 (68.3)	5,506 (72.3)	2,108 (27.7)	< 0.001*

Data presented as number (%)

*P value is below the threshold of ≤ 0.05

Young adults = aged 18–25 years, adults = aged 35–55 years, RYGB = Roux-en-Y gastric bypass, SG = sleeve gastrectomy, *n* = number, T2DM = type 2 diabetes mellitus, GERD = gastroesophageal reflux disease, OSA = obstructive sleep apnea

^*1*^Number of patients with comorbidity status at 1 or 2 years after surgery (patients with postoperative comorbidity status/number of patients with preoperative comorbidity* 100)

## Discussion

Young adults are less likely to undergo bariatric surgery compared to adults, and this might be due to a lack of insight regarding long-term efficacy and safety outcomes [6]. Therefore, this study aimed to increase knowledge on the efficacy and safety of bariatric surgery in young adults using the Dutch National registry. In our study young adults revealed at least comparable weight loss results compared to adults during five years follow-up, both after a RYGB and SG. Moreover, no more postoperative complications and revision procedures were observed. Young adults even had less short-term postoperative complications and more improvement of hypertension, dyslipidemia and musculoskeletal pain.

Bariatric surgery is at least as effective in young adults as in adults with regard to weight loss. Accordingly, a Dutch cohort study revealed similar %TWL up to three years after surgery between young adults (aged 18–25 years) and adults (aged 35–55 years) who underwent a SG and superior %TWL in young adults after a RYGB [22]. This study revealed similar results and even found superior %TWL between young adults and adults up to 5 years postoperatively. Potential reasons for the favorable weight loss in young adults might be the decline in energy expenditure due to a lower resting metabolic rate and a less active lifestyle later in life [23, 24]. Based on our findings, bariatric surgery seems an effective treatment for severe obesity in young adults and even for adolescents as previous literature revealed comparable weight loss results between adolescents and adults [11, 12]. However, not only weight loss is an important outcome measure of bariatric surgery and therefore future prospective studies should also focus on quality of life and monitor the long-term effects of bariatric surgery in this younger age group closely.

The low adherence and follow-up rates of young adults after bariatric surgery have raised concerns. A qualitative study among Dutch young adults who underwent bariatric surgery revealed that young adults find it hard to adhere to postoperative behavioral, dietary and physical recommendations [25]. In line with this, a Swedish national registry study found an increase of missed appointments and loss to follow up in young adults who underwent bariatric surgery compared to adults [13]. Similar results were seen in the five-year follow-up duration of this study. A possible explanation for the lower follow-up rates could lie in the developmental stage the young adults are in. They are more likely to move and go to college compared to adults. Nevertheless, the low follow-up rates of young adults might eventually lead to missed physical or psychological complications. Also, insufficient weight loss might not be detected at an early stage, nor can treatments such as dietary or psychological counseling be provided. A previous study also found that adolescents had more micronutrient deficiencies after bariatric surgery compared to adults, suggesting a lower adherence to multivitamin supplementation [11]. Therefore, clinical practice should focus on optimizing follow-up and compliance rates in the younger age group, for example by adjusting the clinical visits to the need of the young population, e.g. online visits and counseling or group sessions.

Inconsistent findings with regard to postoperative complications after bariatric surgery in young adults are described in pre-existing literature. The Metabolic and Bariatric Surgery Accreditation and Quality Improvement Program (MBSAQIP) database revealed that bariatric surgery is safe in youth and young adults (aged 15–24 years), as low rates of reoperation (1.1%), reintervention (1.1%) and readmission (3.7%) within 30 days were found [6]. A Dutch retrospective cohort study in 130 young adults (aged 18–25 years) observed similar complication rates between adults and young adults up to three years after surgery [22]. In contrast to a large Swedish database study, in which more complications (including serious adverse events (CD 3b-5)) were observed in the young adults (aged 18–25 years) between 6 weeks until 5 years after surgery [13]. This study showed less postoperative complications < 30 days in the young adults group, whereas no differences between the two age groups were found regarding the long-term complications. These differences between complication rates might be explained by study design (cohort study versus database study), subdivision of long and short term complications, definition of young adults, and prevalence’s of preoperative obesity related comorbidities.

The effectiveness of bariatric surgery does not depend on weight loss alone and other factors such as the remission of obesity related comorbidities are just as important. The Teen-Longitudinal Assessment of Bariatric Surgery study revealed similar rates of dyslipidemia remission and more remission of T2DM and hypertension in adolescents compared to adults [11]. In this study, more improvement of hypertension, dyslipidemia and musculoskeletal pain were found in the young adults. Abovementioned results favor bariatric surgery at a younger age, especially as a previous study showed that medical treatments for T2DM fail earlier in youth [26]. Nevertheless, our results should be interpreted with caution as there might be differences in the assessment and registration of obesity related comorbidities among different Dutch hospitals and no long-term data were analyzed.

There are certain limitations to this registry study, inherent to its retrospective cohort design. There might be differences in the registration of data among the different hospitals and errors are likely to occur. Furthermore, some in depth information on operating techniques, obesity related comorbidities, quality of life and physical and psychological complications (e.g. mental health issues, alcohol abuse) are missing in the national database. At last, there was a large loss to follow-up four and five years after surgery and especially in the young adults. This might have caused selection bias, as a previous study revealed that poor weight loss could be a reason for loss to follow-up [27]. However, a sensitivity analysis showed similar weight loss results at three years follow-up between patients with and without four- and five-year follow-up data, rendering large selection bias unlikely.

## Conclusion

Bariatric surgery appears to be at least as safe and effective in young adults as in adults. Compared to adults, even favorable results regarding the improvement of obesity related comorbidities and short-term complications were found in the younger age group. Based on these findings, the reluctance towards bariatric surgery in young adults seems unfounded. Furthermore, clinical practice should focus on optimizing follow-up and adherence rates so that weight regain, physical and psychosocial complications can be prevented or recognized and treated at an early stage .

## Supplementary Information

Below is the link to the electronic supplementary material.

Supplementary file1 (DOCX 16 KB)

## Declarations

All procedures performed in studies involving human participants were in accordance with the ethical standards of the institutional and/or national research committee and with the 1964 Helsinki declaration and its later amendments or comparable ethical standards. For this type of study formal consent is not required.
