# Supplementary material for: A Population-Based Cohort Study on Efficacy and Safety of Bariatric Surgery in Young Adults Versus Adults
Source: Obes Surg. 2023 Jun 26;33(8):2475–84. doi: 10.1007/s11695-023-06673-5 (PMC10344832; doi:10.1007/s11695-023-06673-5)
Supplement: Supplementary file 1 — Supplementary file1 (DOCX 16 KB) [file 11695_2023_6673_MOESM1_ESM.docx]

**Appendix**

**Table 1.** Stratified for bariatric procedure linear mixed model analysis of variables associated with %TWL between young adults and adults until 5 years postoperatively.

|  | **RYGB** | | | **SG** | | |
| --- | --- | --- | --- | --- | --- | --- |
|  | *Beta coefficient* | *95% CI* | *P value* | *Beta coefficient* | *95% CI* | *P value* |
| Age category (young adults vs. adults) | 0.70 | 0.32 – 1.07 | <0.001* | 1.85 | 1.33 – 2.37 | <0.001* |
| Follow-up compared to 1 year after surgery  2 years  3 years  4 years  5 years | 0.33  -1.58  -3.00  -3.90 | 0.25 – 0.40  -1.69 - -1.47  -3.14 - -2.86  -4.08 - -3.72 | <0.001*  <0.001*  <0.001*  <0.001* | -1.06  -3.16  -4.40  -5.45 | -1.22 - -0.90  -3.40 - -2.93  -4.72 - -4.08  -5.91 - -4.99 | <0.001*  <0.001*  <0.001*  <0.001* |
| Follow-up*age category  2 years*young adults  3 years*young adults  4 years*young adults  5 years* young adults | 0.44  0.51  0.28  -0.02 | 0.14 – 0.74  0.07 – 0.95  -0.31 – 0.86  -0.77 – 0.73 | 0.004*  0.024*  0.354  0.963 | 1.10  1.15  0.75  0.07 | 0.71 – 1.49  0.58 – 1.73  -0.06 – 1.56  -1.22 – 1.35 | <0.001*  <0.001*  0.070*  0.920 |
| Sex (male vs female) | -2.56 | -2.82 – 2.30 | <0.001* | -0.40 | -0.86 – 0.06 | 0.087 |
| Preoperative hypertension (yes vs. no) | -0.64 | -0.86 - -0.42 | <0.001* | -1.00 | -1.45 - -0.54 | <0.001* |
| Preoperative T2DM (yes vs. no) | -2.80 | -3.06 - -2.52 | <0.001* | -2.81 | -3.42 - -2.21 | <0.001* |
| Preoperative OSA (yes vs. no) | -0.38 | -0.65 – 0.12 | 0.005* | -0.81 | -1.35 - -0.28 | 0.003* |
| Preoperative dyslipidemia (yes vs. no) | 0.07 | -0.20 – 0.34 | 0.613 | -0.21 | -0.79 – 0.37 | 0.471 |
| Preoperative BMI (kg/m^2^) | 0.14 | 0.12 – 0.16 | <0.001* | 0.07 | 0.04 – 0.10 | <0.001* |
| Postoperative complications > 30 days (yes vs. no) | 1.73 | 1.31 – 2.15 | <0.001* | 2.02 | 0.69 – 3.34 | 0.003* |

**P* value is below the threshold of ≤0.05.

TWL = total weight loss, young adults = aged 18-25 years, adults = aged 35-55 years, RYGB = Roux-en-Y gastric bypass, SG = sleeve gastrectomy, T2DM = type 2 diabetes mellitus, OSA = obstructive sleep apnea, BMI = body mass index.
